# 4-Bromo-*N*-(2-nitro­phen­yl)benzamide

**DOI:** 10.1107/S1600536814003298

**Published:** 2014-02-22

**Authors:** Rodolfo Moreno-Fuquen, Alexis Azcárate, Alan R. Kennedy

**Affiliations:** a Departamento de Química - Facultad de Ciencias, Universidad del Valle, Apartado 25360, Santiago de Cali, Colombia; b WestCHEM, Department of Pure and Applied Chemistry, University of Strathclyde, 295 Cathedral Street, Glasgow G1 1XL, Scotland

## Abstract

The title nitro­phenyl benzamide, C_13_H_9_BrN_2_O_3_, with two mol­ecules in the asymmetric unit, has dihedral angles of 16.78 (15) and 18.87 (14)° between the benzene rings. An intra­molecular N—H⋯O hydrogen bond is observed in each mol­ecule. In the crystal, the molecules are linked by weak C—H⋯O inter­actions; halogen–halogen inter­actions are also observed [Br⋯Br = 3.4976 (7) Å]. These inter­actions form *R* ^2^_2_(10), *R* ^2^_2_(15) and *R* ^6^_6_(32) edge-fused rings along [010].

## Related literature   

For properties of amide compounds, see: Bisson *et al.* (2000 ▶). For the anti­bacterial and anti­fungal activity of amide compounds, see: Aytemir *et al.* (2003 ▶). For similar compounds, see: Moreno-Fuquen *et al.* (2013 ▶); Sripet *et al.* (2012 ▶). For halogen–halogen inter­actions, see: Awwadi *et al.* (2006 ▶); For hydrogen-bonding information, see: Nardelli (1995 ▶). For hydrogen-bond motifs, see: Etter (1990 ▶).
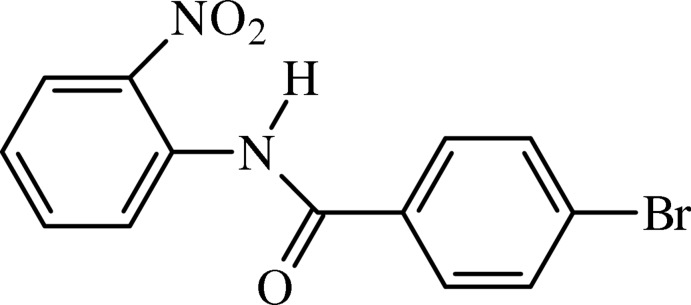


## Experimental   

### 

#### Crystal data  

C_13_H_9_BrN_2_O_3_
*M* *_r_* = 321.13
Triclinic, 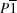

*a* = 3.8338 (4) Å
*b* = 12.6784 (13) Å
*c* = 24.918 (2) Å
α = 81.875 (8)°
β = 88.386 (7)°
γ = 85.460 (8)°
*V* = 1195.1 (2) Å^3^
*Z* = 4
Mo *K*α radiation
μ = 3.45 mm^−1^
*T* = 123 K
0.49 × 0.05 × 0.03 mm

#### Data collection  

Oxford Diffraction Xcalibur E diffractometer
Absorption correction: analytical [*CrysAlis PRO* (Oxford Diffraction, 2010 ▶; analytical numeric absorption correction using a multi-faceted crystal model (Clark & Reid, 1995 ▶)] *T*_min_ = 0.380, *T*_max_ = 0.914
9979 measured reflections
9979 independent reflections
7814 reflections with *I* > 2σ(*I*)

#### Refinement  

*R*[*F* ^2^ > 2σ(*F* ^2^)] = 0.049
*wR*(*F* ^2^) = 0.135
*S* = 1.04
9979 reflections
344 parameters
1 restraint
H-atom parameters constrained
Δρ_max_ = 0.62 e Å^−3^
Δρ_min_ = −0.77 e Å^−3^

### 

Data collection: *CrysAlis PRO* (Oxford Diffraction, 2010 ▶); cell refinement: *CrysAlis PRO*; data reduction: *CrysAlis PRO*; program(s) used to solve structure: *SHELXS97* (Sheldrick, 2008 ▶); program(s) used to refine structure: *SHELXL97* (Sheldrick, 2008 ▶); molecular graphics: *ORTEP-3 for Windows* (Farrugia, 2012 ▶) and *Mercury* (Macrae *et al.*, 2006 ▶); software used to prepare material for publication: *WinGX* (Farrugia, 2012 ▶).

## Supplementary Material

Crystal structure: contains datablock(s) I, global. DOI: 10.1107/S1600536814003298/gg2134sup1.cif

Structure factors: contains datablock(s) I. DOI: 10.1107/S1600536814003298/gg2134Isup2.hkl

Supporting information file. DOI: 10.1107/S1600536814003298/gg2134Isup3.cml

CCDC reference:

Additional supporting information:  crystallographic information; 3D view; checkCIF report

## Figures and Tables

**Table 1:** Hydrogen-bond geometry (Å, °)

*D*—H⋯*A*	*D*—H	H⋯*A*	*D*⋯*A*	*D*—H⋯*A*
C6—H6⋯O4^i^	0.95	2.65	3.378 (5)	134
C16—H16⋯O2^ii^	0.95	2.64	3.349 (5)	132
C19—H19⋯O1^iii^	0.95	2.52	3.262 (5)	135
C3—H3⋯O5^iv^	0.95	2.58	3.299 (5)	133
C23—H23⋯O6^v^	0.95	2.56	3.334 (5)	139
N1—H1*N*⋯O2	0.88	1.92	2.615 (5)	134
N3—H3*N*⋯O5	0.88	1.92	2.628 (5)	136

Symmetry codes: (i) 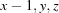; (ii) 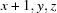; (iii) 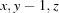; (iv) 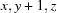; (v) 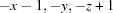.

**Fig. 1.**
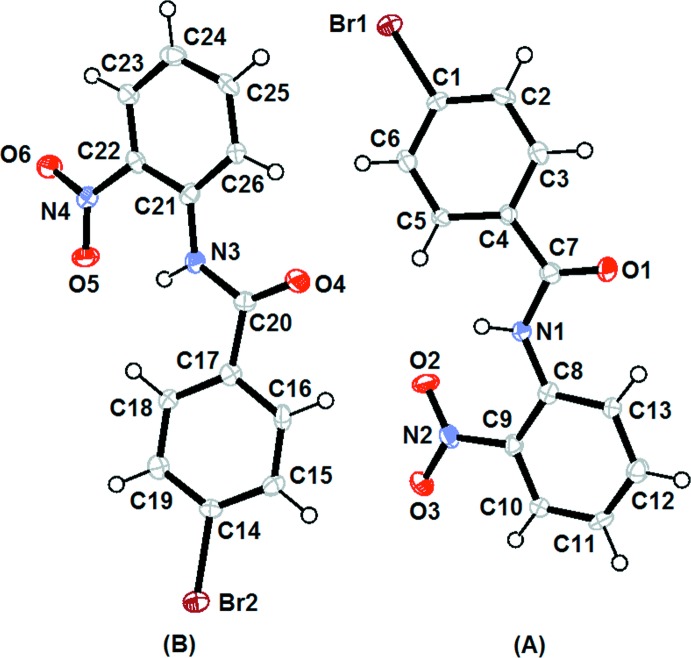
Molecular conformation and atom numbering scheme for the title compound with displacement ellipsoids drawn at the 50% probability level. H atoms are shown as spheres of arbitrary radius.

**Fig. 2.**
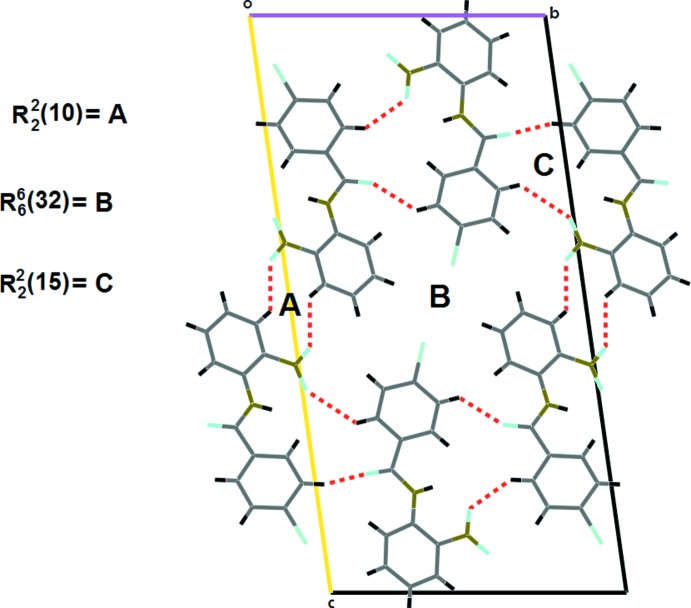
Part of the crystal structure of (I), showing the formation of edge-fused R22(10), R22(15) and R22(32) rings running along [010].

## supplementary crystallographic information

### 2.1. Synthesis and crystallization

The reagents and solvents for the synthesis were obtained from the Aldrich Chemical Co., and were used without additional purification. The title molecule was synthesized using equimolar qu­anti­ties of 4-bromo­benzoyl chloride (0.328 g., 1.495 mmol) and 2-nitro­aniline (0.206 g). The reagents were dissolved in 10 mL of aceto­nitrile and the solution was taken to reflux in constant stirring for 1 hour. Yellow crystals of good quality were obtained after leaving the solvent to evaporate. Yellow crystals of good quality were obtained with m.p of 423 (1)K.

### 2.2. Refinement

Crystal data, data collection and structure refinement details are summarized in Table 1. All H-atoms were positioned at geometrically idealized positions with C—H distance of 0.95 Å and N—H distance of 0.88 Å and U_iso_(H) = 1.2 times U_eq_ of the atoms to which they were bonded.

### 3. Results and discussion

The present compound forms part of a systematic work on N-aromatic amides. The formation of oligomers with properties of molecular zippers have been obtained using molecular templates which include amido ligands (Bisson *et al.*, 2000). Anti­bacterial and anti­fungal activities of different carb­oxy­amide derivatives have been reported (Aytemir *et al.*, 2003). In the synthesis of amides in our group, the 2-nitro­aniline is taken as a template, in order to study the structural changes and the supra­molecular behavior by the reaction of different ligands with this precursor (Moreno-Fuquen *et al.*, 2013). In the reactions involving the 2-nitro­aniline, as precursor, we aimed to synthesize the N-(2-nitro­phenyl)-4-bromo­benzamide (I). A close structure, the 4-bromo-N-(4-meth­oxy-2-nitro­phenyl)-benzamide, (4MNB), (Sripet *et al.*, 2012), has been used as comparison with the structural parameters of the title compound. The title compound has two molecules (A and B) per asymmetric unit (see Fig. 1). The compound exhibits dihedral angles between the benzene rings, very similar: 16.78 (15)° and 18.87 (14)° for A and B molecules, respectively. These dihedral angles are somewhat different when compared with the related compound (4MNB) [2.90 (8)°]. In (I) the other bond lengths and bond angles agree closely with those values presented in its homologous amide (4MNB). The nitro groups form dihedral angles with the adjacent benzene ring of 6.7 (2)° and 9.9 (2)° for O2—N2—O3 and O5—N4—O6, respectively. The crystal packing shows no classical inter­molecular hydrogen bonds and the molecules pack by forming weak C—H···O inter­actions that are propagated along [010] (see Fig. 2). According to the graph-set assignment, the intra­molecular hydrogen-bond pattern generates a S(6) ring motif (Etter, 1990). The crystal packing is stabilized by weak C—H···O inter­molecular inter­actions. The C6 and C3 atoms of the phenyl ring at (x,y,z) act as hydrogen-bond donors to carbonyl O4 atom at (x-1, +y, +z) and to nitro O5 atom at (x,+y+1,+z) respectively. The C16 and C19 atoms of the phenyl ring at (x, y, z) act as hydrogen-bond donors to nitro O2 atom at (x+1, y, z) and to carbonyl O1 atom at (x,y-1,z), respectively. Additionally the C23 atom at (x,y,z) acts as a hydrogen-bond donor to nitro O6 atom at (-x-1, -y, -z+1), (see Table 1; Nardelli, 1995). All these inter­actions form R^2^_2_(10), R^2^_2_(15) and R^6^_6_(32) edge-fused rings along the [010] direction (see Fig. 2). Recent theoretical calculations show that halogen···halogen inter­actions are controlled by electrostatic forces and they display directional character (Awwadi *et al.*, 2006). In the title structure, halogen···halogen inter­actions [Br···Br = 3.4976 (7) Å] within the chains stabilized by C—H···O inter­actions are observed. This Br···Br distance is much shorter than the sum of the van der Waals radii (3.70 Å).

### Crystal data

**Table:**

C_13_H_9_BrN_2_O_3_	*Z* = 4
*M**_r_* = 321.13	*F*(000) = 640
Triclinic, *P*1	*D*_x_ = 1.785 Mg m^−^^3^
Hall symbol: -P 1	Melting point: 423(1) K
*a* = 3.8338 (4) Å	Mo *K*α radiation, λ = 0.71073 Å
*b* = 12.6784 (13) Å	Cell parameters from 4010 reflections
*c* = 24.918 (2) Å	θ = 3.1–28.0°
α = 81.875 (8)°	µ = 3.45 mm^−^^1^
β = 88.386 (7)°	*T* = 123 K
γ = 85.460 (8)°	Needle, yellow
*V* = 1195.1 (2) Å^3^	0.49 × 0.05 × 0.03 mm

### Data collection

**Table:**

Oxford Diffraction Xcalibur E diffractometer	9979 independent reflections
Radiation source: fine-focus sealed tube	7814 reflections with *I* > 2σ(*I*)
Graphite monochromator	*R*_int_ = 0.000
ω scans	θ_max_ = 27.0°, θ_min_ = 3.2°
Absorption correction: analytical [*CrysAlis PRO* (Oxford Diffraction, 2010; analytical numeric absorption correction using a multi-faceted crystal model (Clark & Reid, 1995)]	*h* = −4→4
*T*_min_ = 0.380, *T*_max_ = 0.914	*k* = −14→16
9979 measured reflections	*l* = −31→31

### Refinement

**Table:**

Refinement on *F*^2^	Primary atom site location: structure-invariant direct methods
Least-squares matrix: full	Secondary atom site location: difference Fourier map
*R*[*F*^2^ > 2σ(*F*^2^)] = 0.049	Hydrogen site location: inferred from neighbouring sites
*wR*(*F*^2^) = 0.135	H-atom parameters constrained
*S* = 1.04	*w* = 1/[σ^2^(*F*_o_^2^) + (0.0479*P*)^2^ + 3.1562*P*] where *P* = (*F*_o_^2^ + 2*F*_c_^2^)/3
9979 reflections	(Δ/σ)_max_ < 0.001
344 parameters	Δρ_max_ = 0.62 e Å^−^^3^
1 restraint	Δρ_min_ = −0.77 e Å^−^^3^

### Special details

**Table:**

Geometry. All esds (except the esd in the dihedral angle between two l.s. planes) are estimated using the full covariance matrix. The cell esds are taken into account individually in the estimation of esds in distances, angles and torsion angles; correlations between esds in cell parameters are only used when they are defined by crystal symmetry. An approximate (isotropic) treatment of cell esds is used for estimating esds involving l.s. planes.
Refinement. Refinement of *F*^2^ against ALL reflections. The weighted *R*-factor *wR* and goodness of fit *S* are based on *F*^2^, conventional *R*-factors *R* are based on *F*, with *F* set to zero for negative *F*^2^. The threshold expression of *F*^2^ > σ(*F*^2^) is used only for calculating *R*-factors(gt) *etc*. and is not relevant to the choice of reflections for refinement. *R*-factors based on *F*^2^ are statistically about twice as large as those based on *F*, and *R*- factors based on ALL data will be even larger.

### Fractional atomic coordinates and isotropic or equivalent isotropic displacement parameters (Å^2^)

**Table:**

	*x*	*y*	*z*	*U*_iso_*/*U*_eq_	
Br1	−0.49930 (10)	0.56740 (4)	0.433729 (18)	0.02208 (13)	
Br2	0.96920 (10)	0.05996 (4)	0.084392 (18)	0.02296 (13)	
O1	0.2537 (8)	0.8253 (2)	0.20551 (12)	0.0270 (7)	
O2	−0.0265 (8)	0.4919 (3)	0.14483 (13)	0.0334 (8)	
O3	0.1224 (9)	0.4476 (3)	0.06706 (14)	0.0384 (9)	
O4	0.4069 (8)	0.3367 (3)	0.29067 (13)	0.0313 (8)	
O5	−0.0468 (8)	−0.0126 (2)	0.35060 (13)	0.0312 (8)	
O6	−0.3517 (8)	−0.0460 (2)	0.42413 (13)	0.0285 (8)	
N1	0.1677 (9)	0.6691 (3)	0.17304 (14)	0.0202 (8)	
H1N	0.0934	0.6052	0.1830	0.024*	
N2	0.1123 (9)	0.5117 (3)	0.09994 (16)	0.0229 (9)	
N3	0.2077 (8)	0.1750 (3)	0.32592 (14)	0.0197 (8)	
H3N	0.1732	0.1120	0.3170	0.024*	
N4	−0.1642 (9)	0.0120 (3)	0.39439 (15)	0.0209 (8)	
C1	−0.3041 (10)	0.6174 (3)	0.36484 (17)	0.0168 (9)	
C2	−0.2476 (11)	0.7244 (3)	0.35235 (18)	0.0214 (10)	
H2	−0.3098	0.7727	0.3775	0.026*	
C3	−0.0988 (10)	0.7599 (4)	0.30253 (18)	0.0215 (10)	
H3	−0.0561	0.8331	0.2937	0.026*	
C4	−0.0101 (10)	0.6895 (3)	0.26489 (17)	0.0158 (9)	
C5	−0.0678 (10)	0.5813 (3)	0.27900 (17)	0.0180 (9)	
H5	−0.0041	0.5321	0.2543	0.022*	
C6	−0.2171 (10)	0.5459 (3)	0.32875 (17)	0.0201 (10)	
H6	−0.2598	0.4728	0.3381	0.024*	
C7	0.1510 (10)	0.7357 (3)	0.21253 (18)	0.0194 (10)	
C8	0.2861 (10)	0.6897 (3)	0.11986 (17)	0.0173 (9)	
C9	0.2622 (10)	0.6145 (3)	0.08342 (18)	0.0186 (9)	
C10	0.3722 (10)	0.6340 (3)	0.02913 (17)	0.0194 (10)	
H10	0.3486	0.5824	0.0056	0.023*	
C11	0.5139 (10)	0.7282 (3)	0.01035 (18)	0.0212 (10)	
H11	0.5915	0.7424	−0.0263	0.025*	
C12	0.5432 (10)	0.8028 (4)	0.04532 (18)	0.0228 (10)	
H12	0.6411	0.8682	0.0321	0.027*	
C13	0.4345 (10)	0.7847 (3)	0.09874 (18)	0.0193 (10)	
H13	0.4604	0.8375	0.1216	0.023*	
C14	0.7748 (10)	0.1158 (3)	0.14657 (17)	0.0177 (9)	
C15	0.7605 (10)	0.2246 (3)	0.14817 (18)	0.0200 (10)	
H15	0.8432	0.2718	0.1183	0.024*	
C16	0.6236 (10)	0.2633 (3)	0.19396 (18)	0.0213 (10)	
H16	0.6128	0.3378	0.1957	0.026*	
C17	0.5013 (10)	0.1945 (3)	0.23763 (17)	0.0184 (10)	
C18	0.5160 (10)	0.0848 (3)	0.23493 (18)	0.0193 (10)	
H18	0.4291	0.0375	0.2644	0.023*	
C19	0.6571 (10)	0.0450 (4)	0.18936 (18)	0.0202 (10)	
H19	0.6726	−0.0296	0.1875	0.024*	
C20	0.3678 (11)	0.2434 (4)	0.28646 (18)	0.0194 (10)	
C21	0.0933 (10)	0.1912 (3)	0.37755 (17)	0.0180 (9)	
C22	−0.0783 (10)	0.1120 (3)	0.41268 (18)	0.0187 (10)	
C23	−0.1763 (10)	0.1250 (3)	0.46513 (18)	0.0204 (10)	
H23	−0.2880	0.0701	0.4875	0.024*	
C24	−0.1133 (11)	0.2167 (4)	0.48520 (18)	0.0233 (10)	
H24	−0.1778	0.2254	0.5215	0.028*	
C25	0.0461 (10)	0.2968 (3)	0.45179 (18)	0.0198 (10)	
H25	0.0882	0.3608	0.4654	0.024*	
C26	0.1440 (10)	0.2850 (3)	0.39935 (18)	0.0197 (10)	
H26	0.2486	0.3418	0.3773	0.024*	

### Atomic displacement parameters (Å^2^)

**Table:**

	*U*^11^	*U*^22^	*U*^33^	*U*^12^	*U*^13^	*U*^23^
Br1	0.0215 (2)	0.0273 (3)	0.0174 (3)	−0.00393 (18)	0.00245 (17)	−0.00226 (19)
Br2	0.0207 (2)	0.0277 (3)	0.0208 (3)	−0.00279 (18)	0.00324 (17)	−0.0047 (2)
O1	0.0428 (19)	0.0170 (18)	0.0223 (19)	−0.0113 (14)	0.0046 (15)	−0.0028 (14)
O2	0.052 (2)	0.031 (2)	0.020 (2)	−0.0187 (16)	0.0162 (16)	−0.0100 (15)
O3	0.067 (2)	0.020 (2)	0.032 (2)	−0.0136 (17)	0.0130 (18)	−0.0141 (16)
O4	0.047 (2)	0.021 (2)	0.028 (2)	−0.0090 (15)	0.0118 (15)	−0.0079 (15)
O5	0.049 (2)	0.027 (2)	0.020 (2)	−0.0157 (15)	0.0125 (15)	−0.0091 (15)
O6	0.0359 (18)	0.0234 (19)	0.028 (2)	−0.0123 (14)	0.0114 (15)	−0.0056 (15)
N1	0.029 (2)	0.014 (2)	0.018 (2)	−0.0049 (15)	0.0032 (16)	−0.0033 (15)
N2	0.029 (2)	0.016 (2)	0.024 (2)	−0.0024 (16)	−0.0005 (17)	−0.0049 (17)
N3	0.0251 (19)	0.014 (2)	0.020 (2)	−0.0045 (15)	0.0007 (15)	−0.0029 (16)
N4	0.0220 (19)	0.021 (2)	0.019 (2)	−0.0017 (15)	−0.0012 (16)	0.0000 (17)
C1	0.014 (2)	0.022 (3)	0.014 (2)	−0.0017 (17)	0.0008 (16)	−0.0008 (18)
C2	0.024 (2)	0.023 (3)	0.019 (3)	−0.0006 (19)	0.0036 (18)	−0.010 (2)
C3	0.025 (2)	0.017 (2)	0.024 (3)	−0.0035 (18)	−0.0004 (19)	−0.0044 (19)
C4	0.018 (2)	0.013 (2)	0.016 (2)	−0.0002 (16)	−0.0023 (17)	−0.0016 (17)
C5	0.024 (2)	0.016 (2)	0.016 (2)	−0.0044 (17)	0.0020 (18)	−0.0056 (18)
C6	0.022 (2)	0.016 (2)	0.022 (3)	−0.0012 (17)	−0.0036 (18)	−0.0041 (19)
C7	0.019 (2)	0.019 (3)	0.019 (3)	0.0025 (18)	−0.0001 (17)	−0.0024 (19)
C8	0.013 (2)	0.019 (2)	0.019 (3)	−0.0009 (16)	−0.0022 (17)	−0.0006 (18)
C9	0.020 (2)	0.015 (2)	0.021 (3)	−0.0043 (17)	0.0003 (18)	−0.0019 (18)
C10	0.022 (2)	0.017 (2)	0.019 (3)	−0.0009 (17)	0.0042 (18)	−0.0031 (18)
C11	0.021 (2)	0.029 (3)	0.012 (2)	−0.0021 (18)	0.0040 (17)	0.0013 (19)
C12	0.021 (2)	0.022 (3)	0.025 (3)	−0.0058 (18)	0.0020 (19)	−0.001 (2)
C13	0.023 (2)	0.015 (2)	0.019 (3)	−0.0040 (17)	0.0042 (18)	−0.0027 (18)
C14	0.015 (2)	0.021 (3)	0.017 (2)	−0.0007 (17)	0.0004 (17)	−0.0050 (18)
C15	0.019 (2)	0.023 (3)	0.017 (3)	−0.0059 (18)	0.0026 (18)	−0.0010 (19)
C16	0.021 (2)	0.012 (2)	0.029 (3)	−0.0024 (17)	−0.0003 (19)	0.0019 (19)
C17	0.016 (2)	0.020 (3)	0.020 (3)	−0.0047 (17)	−0.0015 (17)	−0.0037 (19)
C18	0.021 (2)	0.018 (2)	0.018 (3)	−0.0022 (17)	0.0030 (18)	−0.0002 (19)
C19	0.018 (2)	0.019 (3)	0.024 (3)	−0.0033 (17)	−0.0022 (18)	−0.0030 (19)
C20	0.022 (2)	0.020 (3)	0.017 (3)	−0.0017 (18)	−0.0008 (18)	−0.0041 (19)
C21	0.015 (2)	0.020 (3)	0.018 (2)	0.0002 (17)	−0.0015 (17)	0.0011 (18)
C22	0.016 (2)	0.016 (2)	0.025 (3)	0.0016 (16)	−0.0027 (18)	−0.0042 (19)
C23	0.022 (2)	0.019 (3)	0.020 (3)	0.0008 (18)	0.0001 (18)	−0.0035 (19)
C24	0.021 (2)	0.030 (3)	0.018 (3)	0.0067 (19)	−0.0013 (18)	−0.006 (2)
C25	0.020 (2)	0.017 (2)	0.024 (3)	0.0017 (17)	−0.0022 (18)	−0.0094 (19)
C26	0.024 (2)	0.015 (2)	0.020 (3)	−0.0010 (17)	0.0020 (18)	−0.0014 (18)

### Geometric parameters (Å, º)

**Table:**

Br1—C1	1.898 (4)	C9—C10	1.400 (6)
Br2—C14	1.902 (4)	C10—C11	1.368 (6)
O1—C7	1.219 (5)	C10—H10	0.9500
O2—N2	1.227 (5)	C11—C12	1.386 (6)
O3—N2	1.230 (5)	C11—H11	0.9500
O4—C20	1.223 (5)	C12—C13	1.377 (6)
O5—N4	1.239 (5)	C12—H12	0.9500
O6—N4	1.227 (4)	C13—H13	0.9500
N1—C7	1.382 (5)	C14—C15	1.383 (6)
N1—C8	1.385 (5)	C14—C19	1.383 (6)
N1—H1N	0.8800	C15—C16	1.378 (6)
N2—C9	1.467 (5)	C15—H15	0.9500
N3—C20	1.380 (5)	C16—C17	1.390 (6)
N3—C21	1.385 (5)	C16—H16	0.9500
N3—H3N	0.8800	C17—C18	1.399 (6)
N4—C22	1.470 (5)	C17—C20	1.502 (6)
C1—C6	1.381 (6)	C18—C19	1.386 (6)
C1—C2	1.382 (6)	C18—H18	0.9500
C2—C3	1.383 (6)	C19—H19	0.9500
C2—H2	0.9500	C21—C26	1.404 (6)
C3—C4	1.401 (6)	C21—C22	1.425 (6)
C3—H3	0.9500	C22—C23	1.378 (6)
C4—C5	1.400 (6)	C23—C24	1.370 (6)
C4—C7	1.491 (6)	C23—H23	0.9500
C5—C6	1.382 (6)	C24—C25	1.388 (6)
C5—H5	0.9500	C24—H24	0.9500
C6—H6	0.9500	C25—C26	1.374 (6)
C8—C13	1.402 (6)	C25—H25	0.9500
C8—C9	1.415 (6)	C26—H26	0.9500
			
C7—N1—C8	128.5 (4)	C13—C12—C11	121.9 (4)
C7—N1—H1N	115.8	C13—C12—H12	119.1
C8—N1—H1N	115.8	C11—C12—H12	119.1
O2—N2—O3	121.3 (4)	C12—C13—C8	120.9 (4)
O2—N2—C9	120.5 (4)	C12—C13—H13	119.5
O3—N2—C9	118.1 (4)	C8—C13—H13	119.5
C20—N3—C21	129.4 (4)	C15—C14—C19	122.1 (4)
C20—N3—H3N	115.3	C15—C14—Br2	119.6 (3)
C21—N3—H3N	115.3	C19—C14—Br2	118.3 (3)
O6—N4—O5	121.7 (4)	C16—C15—C14	118.6 (4)
O6—N4—C22	117.7 (4)	C16—C15—H15	120.7
O5—N4—C22	120.6 (4)	C14—C15—H15	120.7
C6—C1—C2	121.5 (4)	C15—C16—C17	120.8 (4)
C6—C1—Br1	119.4 (3)	C15—C16—H16	119.6
C2—C1—Br1	119.0 (3)	C17—C16—H16	119.6
C1—C2—C3	118.7 (4)	C16—C17—C18	119.5 (4)
C1—C2—H2	120.6	C16—C17—C20	117.1 (4)
C3—C2—H2	120.6	C18—C17—C20	123.4 (4)
C2—C3—C4	121.0 (4)	C19—C18—C17	120.1 (4)
C2—C3—H3	119.5	C19—C18—H18	120.0
C4—C3—H3	119.5	C17—C18—H18	120.0
C5—C4—C3	118.8 (4)	C14—C19—C18	118.8 (4)
C5—C4—C7	124.4 (4)	C14—C19—H19	120.6
C3—C4—C7	116.7 (4)	C18—C19—H19	120.6
C6—C5—C4	120.1 (4)	O4—C20—N3	123.4 (4)
C6—C5—H5	119.9	O4—C20—C17	121.5 (4)
C4—C5—H5	119.9	N3—C20—C17	115.1 (4)
C1—C6—C5	119.7 (4)	N3—C21—C26	122.7 (4)
C1—C6—H6	120.1	N3—C21—C22	121.8 (4)
C5—C6—H6	120.1	C26—C21—C22	115.4 (4)
O1—C7—N1	123.6 (4)	C23—C22—C21	122.3 (4)
O1—C7—C4	122.3 (4)	C23—C22—N4	116.4 (4)
N1—C7—C4	114.2 (4)	C21—C22—N4	121.4 (4)
N1—C8—C13	122.8 (4)	C24—C23—C22	120.3 (4)
N1—C8—C9	121.2 (4)	C24—C23—H23	119.8
C13—C8—C9	116.0 (4)	C22—C23—H23	119.8
C10—C9—C8	122.5 (4)	C23—C24—C25	119.1 (4)
C10—C9—N2	115.5 (4)	C23—C24—H24	120.4
C8—C9—N2	122.0 (4)	C25—C24—H24	120.4
C11—C10—C9	119.3 (4)	C26—C25—C24	121.2 (4)
C11—C10—H10	120.4	C26—C25—H25	119.4
C9—C10—H10	120.4	C24—C25—H25	119.4
C10—C11—C12	119.4 (4)	C25—C26—C21	121.7 (4)
C10—C11—H11	120.3	C25—C26—H26	119.2
C12—C11—H11	120.3	C21—C26—H26	119.2
			
O2—O2—N2—O3	0.0 (3)	N1—C8—C13—C12	−178.8 (3)
O2—O2—N2—C9	0.0 (4)	C9—C8—C13—C12	1.0 (5)
O5—O5—N4—O6	0.0 (12)	C19—C14—C15—C16	−0.1 (6)
O5—O5—N4—C22	0.0 (13)	Br2—C14—C15—C16	−179.0 (3)
C6—C1—C2—C3	−0.3 (6)	C14—C15—C16—C17	−0.2 (6)
Br1—C1—C2—C3	178.7 (3)	C15—C16—C17—C18	−0.3 (6)
C1—C2—C3—C4	0.8 (6)	C15—C16—C17—C20	178.0 (4)
C2—C3—C4—C5	−1.4 (6)	C16—C17—C18—C19	1.1 (6)
C2—C3—C4—C7	−179.6 (3)	C20—C17—C18—C19	−177.1 (4)
C3—C4—C5—C6	1.5 (6)	C15—C14—C19—C18	0.9 (6)
C7—C4—C5—C6	179.6 (3)	Br2—C14—C19—C18	179.8 (3)
C2—C1—C6—C5	0.4 (6)	C17—C18—C19—C14	−1.4 (5)
Br1—C1—C6—C5	−178.6 (3)	C21—N3—C20—O4	−6.5 (6)
C4—C5—C6—C1	−1.0 (6)	C21—N3—C20—C17	172.1 (3)
C8—N1—C7—O1	−2.2 (6)	C16—C17—C20—O4	−9.4 (6)
C8—N1—C7—C4	176.8 (3)	C18—C17—C20—O4	168.8 (4)
C5—C4—C7—O1	−166.6 (4)	C16—C17—C20—N3	172.0 (3)
C3—C4—C7—O1	11.5 (6)	C18—C17—C20—N3	−9.8 (5)
C5—C4—C7—N1	14.3 (5)	C20—N3—C21—C26	−3.4 (6)
C3—C4—C7—N1	−167.5 (3)	C20—N3—C21—C22	178.0 (4)
C7—N1—C8—C13	4.4 (6)	N3—C21—C22—C23	176.4 (3)
C7—N1—C8—C9	−175.5 (4)	C26—C21—C22—C23	−2.3 (5)
N1—C8—C9—C10	178.5 (3)	N3—C21—C22—N4	−4.3 (5)
C13—C8—C9—C10	−1.4 (6)	C26—C21—C22—N4	177.0 (3)
N1—C8—C9—N2	−0.1 (6)	O6—N4—C22—C23	8.7 (5)
C13—C8—C9—N2	−179.9 (3)	O5—N4—C22—C23	−170.6 (3)
O2—N2—C9—C10	−172.0 (4)	O5—N4—C22—C23	−170.6 (3)
O2—N2—C9—C10	−172.0 (4)	O6—N4—C22—C21	−170.6 (3)
O3—N2—C9—C10	6.2 (5)	O5—N4—C22—C21	10.1 (5)
O2—N2—C9—C8	6.7 (6)	O5—N4—C22—C21	10.1 (5)
O2—N2—C9—C8	6.7 (6)	C21—C22—C23—C24	0.7 (6)
O3—N2—C9—C8	−175.1 (4)	N4—C22—C23—C24	−178.6 (3)
C8—C9—C10—C11	1.1 (6)	C22—C23—C24—C25	0.8 (6)
N2—C9—C10—C11	179.8 (3)	C23—C24—C25—C26	−0.6 (6)
C9—C10—C11—C12	−0.5 (6)	C24—C25—C26—C21	−1.1 (6)
C10—C11—C12—C13	0.2 (6)	N3—C21—C26—C25	−176.2 (3)
C11—C12—C13—C8	−0.5 (6)	C22—C21—C26—C25	2.5 (5)

### Hydrogen-bond geometry (Å, º)

**Table:**

*D*—H···*A*	*D*—H	H···*A*	*D*···*A*	*D*—H···*A*
C6—H6···O4^i^	0.95	2.65	3.378 (5)	134
C16—H16···O2^ii^	0.95	2.64	3.349 (5)	132
C19—H19···O1^iii^	0.95	2.52	3.262 (5)	135
C3—H3···O5^iv^	0.95	2.58	3.299 (5)	133
C23—H23···O6^v^	0.95	2.56	3.334 (5)	139
N1—H1*N*···O2	0.88	1.92	2.615 (5)	134
N3—H3*N*···O5	0.88	1.92	2.628 (5)	136

Symmetry codes: (i) *x*−1, *y*, *z*; (ii) *x*+1, *y*, *z*; (iii) *x*, *y*−1, *z*; (iv) *x*, *y*+1, *z*; (v) −*x*−1, −*y*, −*z*+1.
